# Patterns of structural and sequential ambidexterity in cross-border media management

**DOI:** 10.1080/16522354.2019.1619965

**Published:** 2019-05-30

**Authors:** Pamela Nölleke-Przybylski, M. Bjørn von Rimscha, Johanna E. Möller, Denise Voci, Klaus-Dieter Altmeppen, Matthias Karmasin

**Affiliations:** a School of Journalism, Catholic University Eichstätt-Ingolstadt, Eichstätt, Germany; b Department of Communication, Johannes Gutenberg University Mainz, Mainz, Germany; c Department for Media & Communications Studies, University of Klagenfurt, Klagenfurt am Wörthersee, Austria; d Institute for Comparative Media and Communication Studies (CMC), Austrian Academy of Sciences, Wien, Austria

**Keywords:** Ambidexterity, exploitation, exploration, media management, digitisation, internationalisation

## Abstract

Organisational ambidexterity – the ability of a company to successfully link exploitation and exploration – is a fruitful approach for cross-border management. It is a crucial concept for media companies that, because of the dual (cultural and economic) character of their products, need to reconcile strategies of mere expansion with local customisation when engaging across borders. Drawing on semi-structured interviews with international media managers, this article captures patterns of ambidextrous strategising and organising in cross-border media activities. The article focuses on digitisation, which has altered the opportunities for balancing exploration and exploitation in internationalisation. The analysis reveals how, in this context, exploitation takes centre stage and how patterns of ambidexterity differ significantly depending on the media type and the background of the company.

## Introduction

When venturing abroad, media companies need to reconcile conflicting demands. Cross-border growth ideally provides opportunities for synergy and economies of scale (Chan-Olmsted & Chang, 2003). At the same time, media offerings constituting cultural products (Doyle, 2013) require local adaptation. Thus, an adequate balance of economic standardisation and cultural adaptation is particularly rewarding for the cross-border activities of media companies (Chalaby, 2009; Shrikhande, 2001; Von Rimscha et al., 2018). Working towards such a balance is in accordance with achieving ambidexterity (Han, 2007). An ambidextrous organisation is efficient in managing its current business and is also adaptive to change and diverging market demands (Raisch & Birkinshaw, 2008).

The analytical concept of organisational ambidexterity describes “the ability of an organization to both explore and exploit” (O’Reilly & Tushman, 2013, p. 324). More precisely, the term captures the “delicate trade-off[s] between exploration and exploitation” (March, 1991, p. 85). Exploration and exploitation demarcate various forms of organisational action that appear to work in opposite directions. Exploration and exploitation can be particularised, inter alia, as resource building vs. resource exploitation (Hsu, Lien, & Chen, 2013), innovative vs. adaptive learning (Auh & Menguc, 2005; Benner & Tushman, 2003), diversification vs. focusing (Lavie, Stettner, & Tushman, 2010; Raisch & Birkinshaw, 2008) and flexibility vs. efficiency (Adler, Goldoftas, & Levine, 1999). Ambidexterity stems from the *interconnection* of exploitative and explorative organisational action. It benefits firm performance (Birkinshaw & Gibson, 2004; Cao, Gedajlovic, & Zhang, 2009; March, 1991; O’Reilly & Tushman, 2013) and is crucial for appropriate internationalisation strategies (Han, 2007; Hsu et al., 2013; Johanson & Vahlne, 2009).

Here, we harness the concept of ambidexterity to provide a better understanding of the structures and strategies behind cross-border media activities. Cross-border media activities refer to any activities media companies pursue in foreign territories (e.g. import and export activities, foreign direct investments, or shares in or ownership of companies abroad) (Altmeppen, Karmasin, & Rimscha, 2012; Neubert, 2013). Various researchers have investigated the capabilities and conditions fostering or constraining ambidexterity through cross-border activities (Bandeira-de-Mello, Fleury, Aveline, & Gama, 2016; Cui, Walsh, & Zou, 2014; Hsu et al., 2013; Luo & Rui, 2009; Prange & Verdier, 2011; Vahlne & Jonsson, 2017). Previous research has retraced knowledge transfer and learning processes across borders (Keen & Wu, 2011; Khan, Rao-Nicholson, & Tarba, 2016; Li, 2010) and analysed how products, services and business processes are balanced between global standardisation and local adaptation (Han, 2007). Complementary to these previous analyses, we synthesise multiple definitions of organisational ambidexterity (O’Reilly & Tushman, 2013; Simsek, 2009) to operationalise the concept. Thus, we trace different facets of explorative and exploitative organisational action in cross-border media activities.

Enriching the perspective of previous research, we pay particular attention to the influence of digitisation on the scope and structure of ambidextrous cross-border action. Digitisation might spur cross-border activities, because it accelerates cross-border learning (Osarenkhoe, 2009; Tran, Yonatany, & Mahnke, 2016), facilitates the interlinking of capabilities in home and foreign markets (Coviello, Kano, & Liesch, 2017; Liesch, Buckley, Simonin, & Knight, 2012) and creates virtual marketplaces with lower market entry barriers compared with traditional marketplaces (Autio & Zander, 2016; Grochal-Brejdak & Szymura-Tyc, 2013). Moreover, digitisation potentially blurs the boundaries between local and cross-border businesses (Yamin & Sinkovics, 2006). This is especially applicable to industries and companies whose processes and products are digital or digitised (Kollmann & Christofor, 2014). Media companies are a pertinent example here (Hagenhoff, 2016). Consequently, for these companies, digitisation alters ambidextrous organisational action across borders, for example by easing specific forms of both exploitation (e.g. the scalability of products) and exploration (e.g. cross-border innovation development).

Against this background, we aim to describe patterns of ambidexterity that apply to cross-border media activities in digitised media markets. We use semi-structured interviews with media executives, complemented by document-based organisational analyses to trace the ambidextrous organisational practices of media companies in cross-border contexts. The empirical analysis reveals that digitisation facilitates the particular patterns of ambidexterity applicable to cross-border media activities. Furthermore, patterns of ambidexterity differ by media type and market size. We are able to identify these differences by means of our conceptualisation of ambidexterity by its temporal qualities (structural and sequential) and its operationalisation into particular types and dimensions of organisational action (organising and strategising). Before discussing this conceptualisation, in the following section we explore in more detail the factors that drive exploitation and exploration in cross-border media management.

## (Pre-)conditions of ambidexterity in cross-border media activities

The media industry is characterised by a strong fixed-cost digression and economies of scope (Doyle, 2013); therefore, companies that can draw from economies of scale and synergies have an advantage. This drives concentration and consolidation on national and international scales (Compaine & Gomery, 2000). The exploitative endeavour of scaling beyond national boundaries is moderated by the media’s culture-dependent nature, which necessitates the simultaneous local adaptation of product portfolios and organisational structures (i.e. an explorative approach); however, as will be discussed below, the degree of exploration necessary for geographical diversification varies with media type and with the cultural trade barriers of markets. Additionally, digitisation augments the need for exploration. Parallel to exploring digital businesses, media companies must maintain and ideally foster traditional businesses, in home markets and beyond, because digitisation makes national boundaries more permeable for production and distribution.

### Exploration and exploitation in geographical diversification

Geographical diversification in the media industry is first an exploitative endeavour. Decisions on venturing abroad are guided by the aims of increasing profitability (Oba & Chan-Olmsted, 2007), achieving standardisation (Shrikhande, 2001) and sustaining organisational control over local operations (Strube, 2010). However, geographical diversification in the media industry typically involves exploration on the product level and possibly also on the level of organisational structures. This is because, as audiences prefer local content, media products need to be adapted to the cultural specificities of the target market (Shrikhande, 2001). Irrespective of discussions on media imperialism and the levelling effects of socialisation on cultural discount (Hoskins & Mirus, 1988; Schlütz & Schneider, 2014), cultural properties largely limit the unaltered transmission of media products across borders. As will be argued in the following paragraphs, the degree and particular form of explorative action needed for successful geographic diversification depends on two aspects: (1) the (cultural) characteristics of the foreign market; and (2) the media type. These two aspects are intertwined.

The cultural trade barriers at the market level depend on the size of the home market (Wildman & Lee, 2015), the existence of transnational consumer cultures (Couldry & Hepp, 2012) and, especially, the reach of the company’s native language (Von Rimscha et al., 2018). A common language fosters media exchange beyond national borders (Gershon, 2006), as languages constitute regional clusters of media consumption (Ksiazek & Webster, 2008), production and transfer (Sánchez-Tabernero, 2006). Hence, media companies based in countries with a dominant language (i.e. a language such as English or Spanish that is spoken in a multitude of countries) might easily access a considerably broader market compared with companies based in countries with a non-dominant language (Moran & Keane, 2006). As is also the case for the media’s dependence on societal norms and consumer cultures, the language dependence of the media differs between informative (i.e. prototypically print) and entertainment (i.e. prototypically audio-visual) content. Informative content is produced and consumed predominantly within territorial or linguistic boundaries (Disdier, Tai, Fontagné, & Mayer, 2010), whereas the entertainment sector is largely de-territorialized (Moran, 2009). Publishers typically rely on decentralised structures controlling diverse local entities that produce localised print products (Picard, 2015). For audio-visual entertainment content, the cultural barriers to many target markets are lower or can be overcome by trading customisable formats (Chalaby, 2016). Consequently, the explorative demand is much higher for publishers than for audio-visual companies.

Although all media companies depend somewhat on product adaptation when venturing abroad, not all product adaptability is about exploration. In fact, as media businesses typically engage in a continuous stream of one-of-a-kind production (Küng, 2017), media products that are constantly altered connote exploitative production competencies rather than exploration. The same applies to limited alterations made to media products when they are prepared for cross-border distribution via simple translation or dubbing. Here, geographical diversification might be understood as *exploitation* because it tries to utilise the “firm-specific advantage” (Bandeira-de-Mello et al., 2016, p. 2006). In this sense, Sharma and Blomstermo assert that “[e]xpansion abroad implies transmission of knowledge and domestic based practices. Solutions that have been applied successfully in the past are used […]” (Sharma & Blomstermo, 2003, p. 741).

Similarly, Chan-Olmsted and Chang (2003) have shown that internal factors such as existing alliances or the capability to repurpose content have a significant effect on a media company’s decision to diversify on the geographical and product levels. Chan-Olmsted and Chang did not capture product diversification on the level of the product portfolio but rather of organisational structures. Nevertheless, their results indicate how media conglomerates cope with exploration needs and exploitation opportunities in the context of internationalisation. Media companies thus prefer related diversification and complementary resource alignment, enabling them to benefit from synergies and economies of scale. Media conglomerates combine geographical *and* product diversification. However, recent developments contrast with this result, pointing in the opposite direction: Media companies such as Time Warner Inc., Tegna and Tribune Media focus on particular media types while diversifying geographically (Claussen, 2018; Fitzgerald, 2017).

### Exploitation and exploration in digital (cross-border) markets

To cope with current dynamics in media markets, media organisations need to develop new competencies, adapt existing products or develop new ones, explore new markets and engage in innovative collaboration structures (Küng, 2017), which suggests that exploration could be favoured as a superior strategic option. Whereas exploration is necessary for product innovation in dynamic environments (Jansen, van Den Bosch, & Volberda, 2006), exploitation capabilities are a prerequisite enabling a company to develop exploration capabilities. For instance, exploitation secures a constant flow of income that provides the financial basis for explorative investments (Yalcinkaya, Calantone, & Griffith, 2007). Consequently, ambidexterity appears to be the most promising strategic option, allowing organisations to drive innovation (Andriopoulos & Lewis, 2009; Markides & Chu, 2009) while stabilising and fostering firm performance (Herhausen, 2016; O’Reilly & Tushman, 2013; Raisch & Birkinshaw, 2008). This also applies to media markets (Fojcik, 2015; Järventie-Thesleff, Moisander, & Villi, 2014; Maijanen & Virta, 2017).

Applying ambidexterity is appropriate for multi-platform media companies facing digitisation and technological change because they “need to pursue both incremental and radical change” (Järventie-Thesleff et al., 2014, p. 134) on all platforms at the same time (Maijanen & Virta, 2017). Current market shifts reinforce the dynamics inherent to media markets. At this stage, media firms are confronted with paradoxical challenges – balancing stability, tradition and self-focus with flexibility, change and openness to collaboration – in an environment that questions established industry routines and strategic practices (Horst & Moisander, 2015). Media companies are aware that, to cope successfully with these challenges, they need to combine exploration and exploitation (Fojcik, 2015).

In addition, the digitised economy creates varied opportunities for ambidextrous action in *cross-border* management. Digitisation facilitates the transfer of digitised media products and media components. It promotes knowledge transfer and enables location-independent collaboration (Towse & Handke, 2014). Legacy media companies are forced to (re-)determine the specific value they provide and to decide how to arrange existing resources and competencies while flexibly reacting to market dynamics (Järventie-Thesleff et al., 2014). This is especially applicable for the present topic, as digitisation drives cross-border competition. New media corporations have entered the market with their natively digitised and de-nationalised product portfolios (Birkinbine, Gómez, & Wasko, 2017b). These new media corporations have great financial power and investment capital available for (cross-border) acquisitions. They are also important partners, acting as “media facilitators” for legacy media companies “to explore the new media opportunities” (Chan-Olmsted & Chang, 2003, p. 228). Clearly, exploration in cross-border media markets is increasingly important, and digitisation supports cross-border exploitation because it facilitates the fast and broad distribution of digitised products, enhancing scale (Kollmann & Christofor, 2014).

## Conceptualising ambidexterity

The above literature review provides an outline of the forms of exploitative and explorative action applicable to media companies in a cross-border environment. For a more systematic perspective on these different facets of organisational action, it is expedient to link these types of action to the definitions of exploitation and exploration presented in previous research on organisational ambidexterity. Here, research on both the national and international levels offers valuable insight. We maintain an applicability for cross-border contexts by concentrating on *intra-*organisational and *inter-*organisational settings, leaving aside ambidextrous approaches realised by individual organisational members, as detailed below.

### Strategising and organising in (ambidextrous) organisational action

Ambidexterity does not denote a definite, unidimensional concept; rather, it merges the diverse aspects and elements that characterise exploitation and exploration. We organise these aspects by categorising the different forms of ambidextrous action described in previous research into two dimensions: strategising and organising (see Table 1). Hence, ambidexterity describes organisational structures, but it also refers to the development of strategies. Although “strategy and organisation are fundamentally connected” (Whittington, 2003, p. 122), their analytical separation helps with the interpretation of the particular form and quality of these two facets of ambidexterity. We use the terms organis*ing* and strategis*ing*, instead of organisation and strategy, to highlight our aspiration to delineate “*mechanisms* by which organizations […] strive to achieve ambidexterity” (Simsek, 2009, p. 599). We focus on organisational *practices* rather than static plans and principles.

**Table 1.** Patterns of ambidextrous strategising and organising.

Dimension	Element/Practice	Exploitation	Exploration	Exemplary references
Organising	Resource management	resource exploitation; focus on internal property-based resources	resource building; focus on knowledge-based external resources	Hsu et al., 2013
Organising	Knowledge management and operational structure	knowledge/capability transfer across teams, units, subsidiaries and/or partners	knowledge building in teams and business units; technological and organisational innovation on an inter-organisational level or capability acquisition	Bandeira-de-Mello et al., 2016 Kauppila, 2010 Keen & Wu, 2011
Organising	Organisational learning	adaptive production-oriented and backward-looking experiential learning	product-innovation learning and forward-looking cognitive models	Auh & Menguc, 2005 Benner & Tushman, 2003
Organising	Type of organisational integration	horizontal integration and organisational consolidation	differentiation through vertical and diagonal integration and organisational networks	Gupta et al., 2006 Yamakawa et al., 2011
Organising	Organisational structure	hierarchy	strategic alliance and cooperative structures	Li, 2010
Strategising and Organising	Growth orientation	strategic focus on profit and economies of scale	strategic focus on (explorative) growth	Auh & Menguc, 2005 Han, 2007
Strategising	Degree of diversification	portfolio, segment and geographical focusing	portfolio, segment and geographical diversification	Lavie et al., 2010 Raisch & Birkinshaw, 2008
Strategising	Strategic focus	efficiency	flexibility and effectiveness	Adler et al., 1999 Auh & Menguc, 2005
Strategising	Strategic timeframe	focus on the present, short-term value and profit	focus on the future, long-term success by adapting to long-term changes	Benner & Tushman, 2003 Birkinshaw & Gibson, 2004
Strategising	Market orientation	responsive market orientation reacting to existing demand	proactive market orientation creating demand	Herhausen, 2016

Table 1 lists different types of organisational practices that have been analysed in ambidexterity research and includes a classification of these practices as strategising and/or organising. Media companies focus on the dimension of *organising* when applying exploration or exploitation to organisational learning processes (Auh & Menguc, 2005; Benner & Tushman, 2003; Keen & Wu, 2011), to resource distribution (Hsu et al., 2013) and to networks of interaction within companies and beyond. *Strategising* refers to ambidexterity efforts on the level of corporate strategies, indicating how companies combine the contrasting actions of efficiency and flexibility (Adler et al., 1999; Auh & Menguc, 2005), profit and growth (Han, 2007), short-term value (Birkinshaw & Gibson, 2004) and long-term changes (Benner & Tushman, 2003), and focus and diversification (Lavie et al., 2010).

### Approaches to ambidexterity: contextual, structural and sequential ambidexterity

The literature typically depicts three different ways of combining exploitation and exploration to achieve ambidexterity: *structural, contextual* and *sequential* approaches (O’Reilly & Tushman, 2013; Wu & Wu, 2016). Whereas the sequential approach is based on a successive logic, the structural and contextual approaches imply simultaneous action. Birkinshaw and Gibson (2004) differentiate this simultaneity via the level of the relevant organisational hierarchy. Contextual ambidexterity is achieved when “individual employees divide their time between alignment-focused and adaptability-focused activities” (Birkinshaw & Gibson, 2004, p. 50). These thoughts correspond to the description of ambidexterity as a trade-off between routine and non-routine tasks (Adler et al., 1999). Structural ambidexterity refers to exploration and exploitation “done in separate units or teams” (Birkinshaw & Gibson, 2004, p. 50). Furthermore, structural ambidexterity as specified by O’Reilly and Tushman (2008) “entails not only separate structural subunits […] but also different competencies, systems, incentives, processes and cultures – each internally aligned” (p. 192).

Equivalent to the concept of structural ambidexterity, which links exploitation and exploration in parallel, sequential ambidexterity also “attempt[s] to solve the exploration/exploitation tension through structural means” (O’Reilly & Tushman, 2013, p. 328); however, this is accomplished by temporarily pulling this tension apart. Although temporal separation might also be applicable to the individual level (Gupta, Smith, & Shalley, 2006; Lavie et al., 2010), this is not captured by sequential ambidexterity as applied here. Focusing on the individual level (including the concept of contextual ambidexterity) is neither applicable nor useful for our research for two reasons: First, as O’Reilly and Tushman (2013) point out, the alignment of exploitative and explorative endeavours on the individual level is difficult to trace back to particular “organizational systems and processes” (p. 329). Hence, the mechanisms of organisational ambidexterity remain largely unidentifiable. Second, we focus on *structural* and *sequential* ambidexterity because they are applicable to structures within organisations and, beyond that, to interrelations among various companies. Cross-border activities are strategically and organisationally formed at these two levels of intra- and inter-organisational action (Bartlett & Ghoshal, 1989).

In fact, in an analysis of cross-border activities, the focus is specifically on inter-organisational and intra-alliance relationships and actions. At this level, the ambidexterity discussion explores whether equilibrium is achieved by many companies rather than within a single organisation (Gupta et al., 2006). More specifically, an exploration or exploitation orientation may apply beyond the single organisation to an alliance portfolio (Kauppila, 2015; Lavie & Rosenkopf, 2006; Yamakawa, Yang, & Lin, 2011). Cross-border learning, innovation and technology development merge exploitative and exploratory learning, locating ambidexterity at the alliance level and transcending company borders (Kauppila, 2010; Li, 2010). In this sense, exploration and exploitation can be distributed among partners, subsidiaries or business units in the home and foreign markets (see also Hong & Lee, 2015; Hsu et al., 2013).

### Conceptualising ambidexterity in cross-border media management

Previous research shows that ambidexterity is primarily concerned about how market dynamics are handled and how knowledge generation, learning and innovation processes are organised (Bandeira-de-Mello et al., 2016; Benner & Tushman, 2003; Hong & Lee, 2015; Keen & Wu, 2011; Khan et al., 2016; Li, 2010). Hence, an ambidexterity approach is particularly useful in analysing management in a knowledge-based industry such as media. Further, an ambidexterity perspective captures the interconnectedness of cross-border media activities and digitisation because it reflects how digitisation broadens possibilities for organising processes of learning and innovation development, regardless of national borders (Tran et al., 2016). The definitions and applications of ambidexterity described above serve to refine this perspective for empirical analysis. We synthesise these definitions and applications in the analytical schema displayed in Table 2.

**Table 2.** Conceptualising ambidexterity.

Classifying and specifying forms of ambidexterity …				
… by their **temporal** quality	**simultaneous** pursuit of exploitation and exploration		temporal separation of exploitation and exploration (**successive** logic)	
	contextual ambidexterity	**structural** ambidexterity	**sequential** ambidexterity	
… by the **level** of analysis	media worker	media corporation	media market	
	**intra-organisational**		**inter-organisational**	
	on the level of the individual and the team	on the level of sub-units and organisational divisions	on the level of (formally independent) subsidiaries	on the level of (loose) partnerships, networks and alliances
… by the **dimension** of organisational practices	**organising**: referring to organisational learning, resource distribution, and organisational structure		**strategising**: referring to corporate strategies	
	Organisational ambidexterity	Cross-dimensional ambidexterity	Strategic ambidexterity	

In particular, we consider the differentiation between organising and strategising (see also Table 1) as one analytical category in the classification of ambidexterity. We also harness the differentiation of sequential and structural ambidexterity and the applicability of these two concepts at the level of a particular organisation and its embeddedness into a network of various market players. The three analytical categories – temporal quality, level of analysis, and dimension of organisational practice – can be combined, but they do not constitute an interdependent system. This means, for example, that structural ambidexterity might apply at both the company and the market level, and ambidextrous organising is not reduced to either the intra- or the inter-organisational level. In Table 2, the fields shaded in grey are included in the illustration for the sake of completeness, but they are not relevant to our argument.

## Research design

This article addresses the following main question: Which patterns of ambidexterity apply to cross-border media activities in digitised media markets? Drawing on the analytical specification of ambidexterity as strategising and organising, the first part of the main research question can be specified by the following sub-question:

(RQ 1) Which forms of organising and/or strategising shape the cross-border activities of media companies, and to what extent may these structures and strategies be qualified as ambidextrous?

To consider the influence of digitisation, market size – which is to a large extent influenced by the language of the market – and product characteristics on these forms of strategising and organising, the main research question includes two additional sub-questions:

(RQ 2) How do patterns of ambidexterity differ by the type of media company and the size of the media market?

(RQ 3) How does digitisation constrain or enable ambidextrous patterns in cross-border media activities?

### Sample and data collection

The research question and sub-questions are addressed through semi-structured interviews (Cooper & Schindler, 2014; Patton, 2015) with managers from 24 media companies in Europe and the United States. The interviews were conducted from September 2016 to April 2017 and lasted 71 minutes, on average. The interview language was English or German. Interviewees were asked questions on the type, scope and relevance of cross-border activities; on the strategies regarding products and markets; and on the conditions (e.g. cultural, political, economic) influencing their cross-border efforts. The guideline in the sampling process was to capture maximum variation (Suri, 2011) on several dimensions: type of media and content, company and home market size, and product portfolio. We also captured the traditional background of the media companies because the type of media entails different economic preconditions (Picard & Wildman, 2015) – the materiality of newspapers and magazines results in a higher customising effort if companies want to publish their titles abroad (Picard, 2015).

The sample was selected based on an analysis of trade press coverage and a subsequent investigation of company documents. The informants were senior executives in charge of either cross-border activities or general strategy and business development. Because of their expertise concerning the organisational structures and strategies of their companies’ cross-border activities, the informants were able to provide a bird’s-eye perspective (Blöbaum, Nölleke, & Scheu, 2016). An overview of the companies represented in the sample is provided in Table 3. In the following section, the company numbers presented in Table 3 are used to indicate the sources of all interview extracts.

**Table 3.** Company sample.

No.	Country	Company	Media type
1	AT	ORF-Enterprise	audio-visual content
2	AT	Mediaprint	publishing
3	AT	Austria Presse Agentur	news agency
4	CH	Highlight Communications	film/sports licensing
5	CH	Tamedia	publishing
6	CH	Diogenes	publishing
7	DE	Axel Springer	publishing
8	DE	Vogel Business Media	publishing
9	DE	ZDF Enterprises	audio-visual content
10	DE	Deutsche Presse Agentur	news agency
11	DE	Studio Hamburg	audio-visual content
12	DE	Bertelsmann	multi-media
13	DE	Motor Presse	publishing
14	DE	Hubert Burda Media	publishing
15	EU	European Broadcasting Union	broadcasting
16	BE	De Persgroep	publishing
17	NL	Reed Elsevier	information brokerage
18	UK	ITV Studios	audio-visual content
19	USA	Thomson Reuters	information brokerage
20	USA	Story House	audio-visual content
21	USA	Time Warner	audio-visual content
22	USA	Tribune Content Agency	news agency
23	USA	Time Inc. International	publishing
24	USA	Discovery Networks International	audio-visual content

### Data analysis: capturing ambidexterity and shared strategic patterns

The interviews were transcribed and analysed by means of qualitative content analysis using MAXQDA software. To detect various forms of ambidexterity, we transformed descriptions of explorative and exploitative organisational action found in previous research (see Table 1) into deductive categories. Consequently, we employed deductive coding (Mayring, 2000) to systematically extract information on ambidextrous actions from statements made in the interviews. Where possible, these statements were cross-validated with company information retrieved from company publications and the trade press. We processed these materials via document analysis (Bowen, 2009) and created short summaries of information on the companies’ core business and the cross-border strategies. We integrated these summaries into our analysis using the same coding scheme used for the interviews.

During the coding process, in a process of deductive content analysis, we first captured the company-specific perspective on exploitative and explorative strategising and organising. Next, we consolidated the results for companies across different company types, following a cross-case analysis logic (Burns, 2010): Because we had assigned pertinent variables to our material, we were able to detect differences and commonalities in our codifications among companies, across media types and in different language markets. Based on this consolidation of the results, we reprocessed the material. In doing so, we inductively developed categories to further classify commonalities and to rearrange our results (Kuckartz, 2014). In particular, we identified patterns of sequential and structural ambidexterity with similarities in strategising and differences in organising, depending on the media company’s type and national background. In the following section, we present the results of this second cross-unit step (Gerring, 2004). Our methodological approach (deductive coding followed by inductive restructuring) led to findings that are not structured based on our initial conceptualisation but rather along the identified patterns.

## Results: patterns of structural and sequential ambidexterity

Strategy designs and organisational structures detected for particular media companies reveal both common and distinctive patterns of ambidexterity. Here, we focus on the *strategic* dimension, identifying “pruning” and “coring” as two mechanisms media companies use to harness digitisation. The second subsection substantiates how these two strategic preconditions for cross-border activities promote a global view – an aspiration towards and focus on generic global markets rather than particularised local markets (Bartlett & Ghoshal, 1989), resulting in structural and sequential ambidexterity. This highlights exploitative and explorative company activities with a specific focus on the *organisational* dimension. The third subsection details how media companies from minor-language countries emphasise exploitative endeavours.

### Pruning and coring: enhancing concurrent exploitative and explorative action

**Figure 1.**
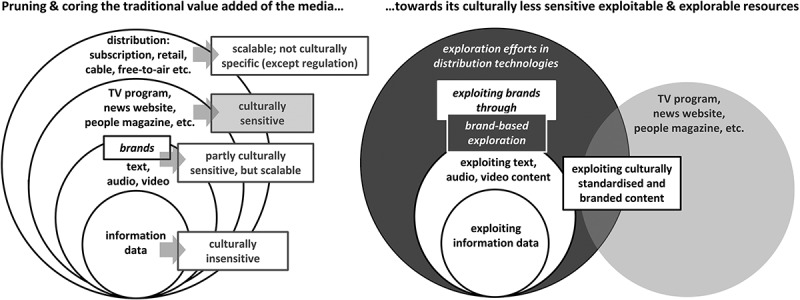
Broadening the scope for exploitative and explorative action.

The interview analysis reveals how digitisation fosters a basic tendency that many media companies aspire to when venturing abroad. For cross-border activities, media companies increasingly concentrate on immaterial product elements that are scalable and not culturally sensitive. Specifically, venturing across borders is less about wrapping media content into traditional packages (i.e. media products such as a television programme or a printed magazine) than about focusing on the core of value creation – the unbundled content independent from its traditional medium. The exploitable value of this content resides in two facets. The first is the particular information processed into diverse formal incarnations (i.e. text, video, audio and pictures) and the resources generating these information packages. The second is the brand lending credibility to the content in the marketplace. Media companies harness these two exploitable facets of media content by applying the strategies of “pruning” and “coring”. Pruning is the process of directing the focus onto the product components, resources and production competencies. Coring is the removal of the traditional product core to exploit the brand as a cored frame. Pruning and coring both facilitate exploitative and explorative endeavours. Figure 1 illustrates this development.

#### Pruning in publishing: re-focusing on a scalable business

Media companies *re-*focus on what they know best to transcend borders and grow easily. The possibilities for growth in the sense of scaling accrue from the process of re-defining the core of value creation – stepping back (within the value-creating process) from the step of producing particular media products:

In the past, we thought […] we are promoting magazines and distributing printed matter and then, basically, we noticed due to this pressure by the Internet that the core of our business is in fact collecting knowledge […]. [8]

This extract from the German specialist publisher Vogel Business Media illustrates how they are pruning their business portfolio into the core of value creation. Thereby, the re-definition of a company’s profile by means of pruning is bound to investments in digital businesses, particularly in new, digital means of distribution. An executive of Time Inc. International made the following statement:

[…] we’re taking what used to be a publishing business and […] you could say transforming it into a digital business. […] It’s […] transforming it into a multiplatform distribution […] business. [23]

Explorative efforts support the (new) emphasis of publishers on products that are both digital and less culturally sensitive. These immaterial businesses are scalable and less affected by borders. They facilitate exploitation because they enable publishers to transcend the need for cost-intensive geographical diversification. Eventually, digital businesses inherently qualify for cross-border transfer: “Internationalisation […] takes place in digitisation” [8]. For instance, German special-interest publisher Motor Presse is distributing its fitness app in 120 countries: “Basically, we retreat on the scalable business – in principal, a digital behaviour. At the same time, we are entering new countries with digital businesses” [13]. Similarly, Belgian publisher De Persgroep highlighted its effort of strengthening its digital business through cross-border expansion and conversely expand its cross-border activities through digitisation: “As our future will be more and more digital, our future will be more and more cross-border” [16]. This tendency illustrates how cross-border distribution and digitisation are two mutually dependent market trends. Companies engaging in information brokerage prototypically represent the interrelation of digitisation and global reach: Thomson Reuters and Reed Elsevier digitally source and distribute professional information. Their offerings are – despite their selective local character – highly scalable.

#### Coring: leveraging brands for structural ambidexterity

Legacy media brands are a crucial resource for cross-border activities because brand recognition provides value, trust in the product and reputation. German special-interest publisher Vogel Business Media cooperates with a German partner in India “because of our name, because of our brand […]” [8]. The media brand can be regarded as an internal property-based resource that lends itself to exploitation. At the same time, it is the starting point for exploration on the product level. Hence, brands are paving the way for an ambidextrous approach. They do so in two ways. First, the brand in its traditional form sustains new ventures. Motor Presse needs its magazine business “to fuel our events and to have an offline brand accompanying our digital offerings, because print still has a lead in credibility” [13]. Second, media companies also develop new products by applying their brands to new product types such as events.

Actually, I think that, whether it’s video, web content or content that travels into printed forms, […], we’re really being very creative and very aspirational, and any and all things that we can do with the brands. [23]

Cross-border product diversification indicating exploration primarily but not exclusively occurs via brand applications. These results support research on media brand management that describe internationalisation as a brand extension (Doyle, 2015). Complementarily, media companies explore on the level of distribution: They invest in technological innovation and distribution technologies, trying to expand their ability to transmit and thus scale their brands. The aim is to create a ”distribution business leveraging our brands and our content” [23]. “Leverage” [21] is a key word in cross-border brand application that is also referred to by audio-visual companies. However, for this media type, the brand is not being cored; rather, the brand value (e.g. of a particular movie) is established by the particular content itself. A strong content brand “tremendously helps you in marketing” [4]; for example, brands such as the Eurovision Song Contest “unite an audience of millions, and they survive anything” [4]. For companies engaging in audio-visual production and distribution, sport rights assume a similar function. Consequently, cross-border brand application as pursued by audio-visual companies is often exploitative, whereas publishers are more explorative. One exception to this rule constituting the basis for ambidextrous action are formats for audio-visual programming. These formats combine efficiency (exploitation of the centralised format branding and production knowledge) and flexibility (cultural adaptation through decentralised format-based production). As such, they can be “multiplied everywhere” [12]. Hence, for British audio-visual production company ITV Studios, “our [format] brands are our currency” [18].

Clearly, brands are opening doors in foreign countries for both publishing and audio-visual companies. Brands facilitate cross-border explorative activities, but it is an exploitative endeavour that most significantly drives their application. The following section substantiates this argument.

### A global view: patterns of structural and sequential ambidexterity

Pruning offerings and leveraging brands while concurrently investing in digital (production and distribution) businesses promotes a global view whereby these strategic orientations enhance geographical diversification. This predominantly applies to companies in dominant-language countries that are oriented towards scale. However, particular companies from non-dominant-language countries (publishers engaging in special-interest content production and distribution) can also apply pruning and coring strategies.

Against this background, cross-border activities are characterised by ambidextrous action. The pattern of exploiting product resources through product portfolio exploration activities (e.g. by applying a publishing brand to video production) is a form of *structural ambidexterity* because exploration and exploitation are occurring simultaneously. Additionally, the exploration of digital businesses is a further step towards scaling. For instance, audio-visual corporations invest in cross-border vertical integration, expanding their capabilities in distribution aiming at economies of scale. Consequently, most companies harness digitisation for *sequential ambidexterity*, but with a pro-profit focus; ultimately, the emphasis is on exploitation. German publisher Axel Springer, for example, has been expanding quite aggressively in digital businesses and now aims at exploitation by focusing. The acquisition-based phase of expansion has resulted in an “awareness of having a lot of great trump cards at our disposal which we also have to use now. You cannot take care of a new project daily” [7].

A closer look at the organisational practices that constitute both sequential and structural ambidexterity reveals variation between types of media companies. Two different patterns of organising the relationship between headquarters and local entities distinguish publishers from audio-visual companies: Publishers retrench their local activities, whereas audio-visual companies de-localise resources built via local investments. Print media companies tend to withdraw from decentralised structures if possible. This is a development that is “completely against the culture” [13] of publishers, who traditionally thought in decentralised structures. For instance, Motor Presse is slowly withdrawing from its local subsidiaries, aiming to restructure their cross-border activities into scalable licensing businesses. However, this does not imply that these companies are not aware of the importance of local knowledge. In fact, the opposite is true:

I think we know what we know, and you have to know what you don’t know. […] So, our view is we find like-minded high-quality partners and look to work with them in individual markets. […] And we don’t have the capacity to run smaller businesses that really don’t scale. So, they won’t scale for us, but they’ll scale for a local player. [23]

Thus, local knowledge is a precondition for local success; however, from the perspective of the company as a whole, rewarding cross-border activities is about scaling. Loose partnerships become increasingly important because owning and operating structures would not scale. Hence, decision making is centralised, and local knowledge is acquired through local alliance partners. Still, localised activities also serve to re-transfer locally acquired knowledge and resources.

This is particularly true for audio-visual companies and already sketches the relationship pattern typical for this media type. Whereas publishers use central assets, seeking to peripherally explore and capitalise them with the help of local partners, audio-visual companies use decentralised structures for exploitation, using investments and shares in local distribution and production units to exploit existing content brands while also generating local assets that might be centrally exploited. This means that some audio-visual companies invest in expanding their peripheral infrastructure. They do so “to capitalise into IP [intellectual property], into formats that may be developed anywhere” [21]. Local entities are motivated to think globally. A Time Warner executive explicated the role of local television and film production units for the company as a whole as follows: “It is less about developing local scripts, and it’s more about taking concepts that are very global in nature” [21]. Similarly, a representative of ITV Studios made the following remarks:

We have a lot of different independent production companies that sit under the ITV Studios umbrella. They have different specialisms. […] We would prepare them for the market, and then we would push them out to the international studios and to the other territories. […] So, we suck up that information, prepare it for market, push it out to everywhere including the U[nited] K[ingdom]. [18]

These interview extracts may hint at a tendency towards the de-nationalisation of local subsidiaries. The categorisation of both activities and products (resulting from these activities) by *national* markets might become less important. This is in contrast to the focus on local positioning as set by media companies from countries with smaller, non-English-language home markets.

### A local focus: emphasising exploitation in small (language) markets

The strategies that have been described to this point in the article broaden the scope for cross-border scaling through either levelling cultural dependence or delegating the need for cultural customising to local partners. These two strategy components are also often combined. Interestingly, these patterns do not apply to most media companies based in minor-language markets, whose cross-border actions reveal an emphasis on exploitation efforts and the core of value creation by largely sticking to the *traditional* business. In cross-border markets, these companies focus on what they know best (i.e. traditional segments and products). They do not engage in exploitation via scaling but via efficiency, niche and segment diversification strategies. These findings are applicable across different media types, applying equally to newspaper publishers, news agencies and audio-visual companies.

For media companies, being located in a minor-language market usually implies a smaller home market and a constricted scope for cross-border action. These companies face a greater need for product customisation if they want to export their media products. They might broaden their scope of action by applying a digital business logic (i.e. by developing scalable businesses). Some companies from minor-language countries aspire to do this. However, a closer look shows that these scalable businesses are not media businesses in the traditional sense but are rather, for example, investments in technologically oriented start-ups [2] or joint ventures developing digital services [3], [10]. The traditional media business is not scalable: “I don’t see this scalability with news from Austria. […] with niche products but not with a general news blog” [2]. The CEO of Burda International highlighted the local character of the business, which calls for localised production units in different countries and for the customisation of content. Although media business is local in general – “all business is local” [10] – this is especially true for publishing. Publishing brands epitomise a national heritage:

[…] you’re talking about brands that have a heritage of 50 or 100 years – even more years of history in their culture […]. We don’t think that, when you go to the end consumer, that there’s something like a cross-border or one market, even if we speak the same language. [16]

Local sales presuppose the existence of a local editorial staff, as a representative of Vogel Business Media explained: “Simply translating and then exporting [the magazines] into the country does not work. We always had and we still have [our] own editorial offices in the countries” [8]. However, maintaining local subsidiaries is costly, and revenues from advertising are in decline. Thus, the sustained need for local customisation renders cross-border activities in traditional publishing economically unattractive. Going international has thus lost its priority. It is not a goal in and of itself, a strategic orientation that was also stressed by De Persgroep:

We bought some magazines in Belgium from Sanoma. […] And then we said, ‘That will be our last acquisition in print’. We were consolidating the market. […] [But] never say never. If a good print add-on acquisition that brings more scale became available, we wouldn’t say no if it’s at a good price. [16]

As this extract indicates, future acquisitions might occur if they facilitate scale for the overall business. Thus, scale is not about global expansion, but rather geographically restricted cross-border penetration. Without an opportunity to reach critical scale, local units tend to be inefficient. Austrian newspaper publisher Mediaprint explained this as follows: “[…] we had shares in free newspapers abroad that we have sold by now. We found that you cannot serve foreign markets with small units” [2].

A recurring pattern for companies from minor-language markets is an emphasis on core activities with cautious diversification of product portfolios. They grow via horizontal integration and tend to strengthen their existing customer relationships. For Swiss publisher Tamedia, cross-border activity is about synergising to achieve more efficiency: “[Our] strategy is also to try to do what we are doing well” [5].

If it occurs at all, diversification happens in regard to segments rather than products. For instance, German news agency DPA now caters to non-media customers with what they know how to do best wrapped into content marketing. Basically, companies from minor-language markets tend to concentrate on specific geo-lingual markets and small markets where competition is less fierce. DPA and its Austrian counterpart, APA, for example, rely on a neat cooperation in the German-speaking region: “We do have the most [cooperation] in the so-called D-A-CH^1^ region. This is because we share the same language” [10].

There is still potential for the exploitation of cross-border markets. Some companies highlight their specific cultural knowledge and cultural sensitivity. Although this can limit diversification options, it can also better position foreign markets. An executive at ORF Enterprises, the international sales house for the Austrian public service broadcaster, made the following argument:

You mustn’t diversify too much because this would not make sense with regard to cost–benefit ratio. […] This is why we are focusing on Austrian productions […], because this is a unique selling proposition. [1]

An executive at Story House, a production company rooted in both Germany and the United States, highlighted the idea that, although narrative styles are often specific to particular national markets, they can still be related to a common denominator at regional level (e.g. the European market). This creates the possibility of producing cost-effective local adaptations “based on a modular concept” [20]. In this way, the company can maximally exploit the same material. The process of customising content occurs with the help of local partners who are familiar with the national nuances of narration styles. As a result, the cross-border activities of Story House have an ambidextrous character because they are about segment diversification while using existing capabilities. Applying a more exploitative approach, German television producer Studio Hamburg engages across borders to gain additional revenues with products initially produced for the German-language market: “[…] clearly, we attempt to generate additional revenues for our product – to refinance the product” [11]. Moreover, cross-border co-production is seen as risk reduction, rather than an opportunity for strategic resource building.

### Summary of results: patterns of ambidextrous organising and strategising

An analysis of the cross-border activities of media companies reveals that there is no “gold standard” when strategically and organisationally designing these activities. However, using the concepts of sequential and structural ambidexterity (O’Reilly & Tushman, 2013) as an analytical lens, we were able to identify generalizable patterns applicable in cross-border media management. Table 4 sketches the overarching patterns of ambidexterity and the forms of strategising and organising that lead to these patterns (see RQ1). As has been described in the previous sections, three types of media companies ideal-typically differ in terms of which of these patterns are applied (see RQ 2). These media company types are (1) publishers targeting the global media market; (2) audio-visual companies adopting a global orientation; and (3) media companies (of different media types) located in non-dominant-language countries. Organising and strategising induced by digitisation is traceable across all three types, with the most significant enabling effect observed for publishers targeting the global media market (RQ 3).

**Table 4.** Patterns of organising and strategising resulting in ambidextrous action.

	Global view » Publishers	Global view » Audio-visual companies	Local Focus » Non-dominant-language countries
patterns of organising	withdrawing from decentralised structures expanding cross-border activities by means of centralised, digital businesses or harnessing decentralised partnership networks headquarter directs knowledge transfer	exploiting centralised assets (e.g. distributing United States content locally) horizontal integration for (1) exploiting assets; (2) generating assets that might be centrally exploited (formats) vertical integration (into rights trade and distribution)	exploiting knowledge-based resources (e.g. self-concept as a niche provider) knowledge building and investment in digital/IT withdrawing from local units or replicating the business abroad
patterns of strategising	re-focusing on the core of value creation, i.e. (1) digital/immaterial content; (2) brand segment focusing: special-interest content facilitates scaling geographical diversification with scalable products	focus on audio-visual business diversifying into (audio-visual) distribution (over-the-top (OTT), streaming, apps) balancing efficiency and flexibility in format trade and development	focus on existing business restricted geographical diversification: focus on a limited number of markets emphasis on efficiency; hope to build some scalable business: reactive approach
			
Corresponding pattern of ambidexterity	**structural ambidexterity** *with a focus on exploitative strategising*: pruning and coring to provide a basis for further exploitation of existing resources by particular exploration activities *explorative organising* takes place as **structural ambidexterity** and aims at **sequential ambidexterity**: explorative investments in digital businesses to generate a basis for a scalable business	**structural ambidexterity** *in both organising and strategising*: format trade **sequential ambidexterity** *via ambidextrous organising*: investing in decentralised structures that provide centrally exploitable assets *explorative organising induces* **structural ambidexterity** and aims at **sequential ambidexterity**: explorative investment in digital distribution to generate a basis for a scalable business	*focusing on efficiency and* **exploitation**: cross-border activity is in itself explorative and therefore expensive – tendency to foster existing infrastructure/resources aiming at **sequential ambidexterity**: cautious explorative investment in digital businesses to generate a basis for a scalable business

Patterns of structural ambidexterity apply to both publishers and audio-visual companies adopting a global perspective (i.e. aiming at geographical expansion). Patterns of sequential ambidexterity identified for all company types entail one specificity: They are each characterised by a sequentiality of exploitation following exploration. This resembles a finding reported by Bandeira-de-Mello et al. (2016) in their analysis of a Brazilian multi-national company: “Exploitation creates value through already developed competences, following successful exploration” (Bandeira-de-Mello et al., 2016, p. 2006). Hence, exploitation through cross-border activities not only characterises companies based in non-dominant-language countries, but is also implicitly emphasised in all of the patterns of sequential ambidexterity that we identified.

## Discussion

Complementing previous research that hints at how digitisation facilitates internationalisation (Autio & Zander, 2016), our results show that digitisation is both a means to and a goal of cross-border activities. It is easier to spread digital than physically bound media content across various markets – at least if it is disburdened from cultural-sensitivity aspects that would require customisation. Indeed, scalability is associated with product standardisation (Kollmann & Christofor, 2014). Hence, digitisation in cross-border media management initiates explorative investments in distribution capabilities but ultimately fosters exploitation.

Cross-border diversification of product portfolios occurs in a cautious way and relies on loose partnerships. Thus, *ambidextrous organisational tendencies* guide media companies’ cross-border activities. The openness to exploration at first sight contrasts with the attractiveness of exploitation, which provides short-term but certain and immediate value (Benner & Tushman, 2003; Kauppila, 2015; March, 1991). However, for media businesses, exploration is an unavoidable reaction to the pressure exerted by the dynamics of a digitised market. The strategic focus behind explorative organising substantiates this assumption. The underlying strategy is very much exploitative: *Strategic ambidexterity* tends towards strategic exploitation.

Such an emphasis on exploitative strategising might transform ambidexterity from a long-term solution into a mere vehicle for an exploitative endeavour. This endangers both intra- and inter-organisational ambidextrous activities because they do not substitute for, but rather complement, each other (Kauppila, 2010). At the same time, as traced in the media-specific pre-conditions, an ambidextrous approach is vital to media companies because it secures a combination of cultural adaptation and economic standardisation in cross-border media management. Moreover, ambidexterity represents a sustainable reaction to digitisation (Järventie-Thesleff et al., 2014; Maijanen & Virta, 2017). Hence, media businesses stressing exploitation might have both economic and societal consequences.

An exploitative focus might cause media companies to lose their openness towards exploration and therefore also their flexibility. Ongoing technological changes will oblige media companies to engage continuously in technological development. As a result, their former core values might become no more than a vehicle for value creation, overturning the previous relationship of production and distribution: Distribution becomes more important than production. In fact, although it might have been reasonable to perceive cross-border activities in distribution as riskier than content creation at the beginning of the millennium (Chan-Olmsted & Chang, 2003), these risks may no longer apply to digital distribution. After all, investments in distribution are no longer geographically specific, and thus they are no longer connected to geographical or regional risks. However, national regulations and nationally varying technological infrastructures constitute new boundaries for distribution activities (Moen, Gavlen, & Endresen, 2004).

The production process highlights the cultural and social value of media, whereas the process of distribution represents an economic focus (Altmeppen, 2006). Consequently, if media companies gradually withdraw from culturally sensitive products, they incrementally stress an economic rather than a cultural/publicist logic. Hence, they are potentially destabilising the societally important core of media work. Insofar as cultural products are homogenised for wider exploitation, this affects culture because “media giants contribute to the commodification of culture” (Birkinbine, Gómez, & Wasko, 2017a, p. 481).

Our approach provides a differentiated look at these tendencies. We identified differentiated patterns of ambidexterity via our categorisation of organisational ambidexterity into particular organisational actions. Hence, our operationalisation results can distinguish different forms of ambidexterity that would appear to denote similar phenomena at a more abstract level. Moreover, in addition to major media companies, this approach can also be applied to so-called “second-tier” media companies, which are worthy of close consideration but are neglected in transnational media management research (Gershon, 2006). Still, despite the value of a focus on particular organising and strategising practices, a coherent interpretation is only possible by considering every company as a whole. An emphasis on either exploitation or exploration does not stem merely from an aggregation of pertinent codifications. Future research should consider the particularity of each media company to classify its strategies adequately and to evaluate sufficiently whether these strategies thwart its societal role.

There are several limitations in our research that stem from our process of comparing a limited number of media companies from a particular sample of countries. Primarily, the national markets considered in our study represent different language markets but have limited differences in terms of culture (Hofstede, 2003). Similarities among companies based in different countries might result from commonalities in Western management thought. By considering media companies based in Non-Western countries, researchers might capture managerial solutions that deviate from Western thinking but that are still applicable to Western markets and may even be superior to existing solutions. Furthermore, higher validity in describing ambidextrous action might be reached by comparing cross-border management at the level of activities instead of at the company level (Möller et al., 2019). In fact, companies pursue diverse, sometimes contrasting, activities. Particular patterns of organising and strategising may be more appropriately applied to particular activities than to a company as a whole. However, as argued above, the company as a whole remains central in interpreting the results. Finally, as highlighted in our approach, organis*ing* and strategis*ing* highlight a process dimension, but our research design captures management only at a single point in time. Longitudinal studies could explore how ambidextrous action develops. Moreover, these types of studies would be able to capture the de facto sequential ambidexterity instead of mere plans that still need to be enacted in managerial reality.

## Note

1. D-A-CH is an abbreviation for the three neighboring German-speaking countries Germany (D), Austria (A) and Switzerland (CH).
